# A multidimensional-scaling study of images from diverse everyday-object categories

**DOI:** 10.3758/s13428-026-02987-1

**Published:** 2026-04-16

**Authors:** Robert M. Nosofsky, Adam F. Osth

**Affiliations:** 1 Department of Psychological and Brain Sciences, Indiana University, 1101 E. Tenth Street, Bloomington, IN 47405 USA; 2 Melbourne School of Psychological Sciences, The University of Melbourne, Parkville, Australia

**Keywords:** Multidimensional scaling, Similarity, Old–new recognition, Everyday-object categories

## Abstract

We propose and implement an approach for deriving multidimensional scaling (MDS) solutions for objects from diverse everyday-object categories. The goal is for the MDS solutions to capture relative similarities between pairs of objects both within and across the categories. For example, if the members of the category *apples* are more similar to one another than are the members of the category *lamps*, then the MDS solution for the apples will be more compressed overall than the MDS solution for lamps. To achieve this goal, the key idea is that, rather than collecting similarity-judgment data one category at a time, we alternate in random fashion across trials the category from which the similarity-judgment data are collected. We hypothesize that if similarity-judgment data are collected one category at a time, observers may recalibrate their judgment scale with respect to each individual category, which could cause loss of information of overall discriminability relations across the different categories. By using the alternating-category approach, observers may be able to maintain a more nearly constant judgment scale across the different categories. We combine the alternating-category procedure with the use of metric forms of MDS that produce MDS solutions in which differences in overall discriminability relations across categories are maintained. We provide preliminary evidence of the success of the approach by showing that, when used as input to a simple computational model of recognition memory, the derived MDS solutions predict reasonably well the false-alarm rates associated with the different categories observed in an old–new recognition experiment.

Research in cognitive science often requires the use of stimuli with varying degrees of similarity to one another. Although much research has made use of highly simplified perceptual stimuli for which interitem similarities can be precisely measured, a more modern trend has the goal of investigating and developing computational models of performance in real-world domains involving complex stimuli. Measuring similarities among real-world high-dimensional stimuli is a challenging task.

One example of a stimulus database involving images of real-world objects that has seen widespread applications is the “Massive Memory” database, first introduced in the classic studies of Brady et al. (2008) and Konkle et al. (2010). The portion of the database of central interest in the present study provides 16 or 17 images from 240 everyday-object categories. We are currently engaged in a project that seeks to test alternative computational models of old–new recognition memory in experiments that make use of a subset of the items from this massive database (Osth & Nosofsky, in preparation). We recently accomplished something very similar in another high-dimensional stimulus domain, namely geologically defined categories of rock images (Nosofsky et al., 2025). Applying the computational models in such domains requires that the items be embedded in psychological spaces providing information about the similarities among the individual items (e.g., Nosofsky, 1992; Roads & Love, 2024).

Thus, our goal in the present work was to derive multidimensional scaling (MDS) solutions for members of a subset of categories from the Massive Memory database. In MDS, each object is represented as a point in a multidimensional space, and the psychological similarity between objects is assumed to be a decreasing function of the distance between the objects in the space (Hout et al., 2012; Lee, 2001; Shepard, 1962, 1980). These MDS solutions could then serve as a source of input for the alternative recognition-memory models tested in our project.

As explained in more detail below, a critical goal in deriving the MDS solutions was that in addition to capturing the structure of similarity relations among objects within each of the individual categories, the derived solutions preserve information about how pairwise similarities compared *across* the different categories. For example, if the psychological similarities among members of the category of *apples* tended to be greater than those among the category of *backpacks*, then the MDS solution for the apples would be more compressed than that for backpacks. As we argue below, for the present database of highly diverse categories, this goal may be a difficult one to achieve, for multiple reasons.

In previous work, Hout et al. (2014) engaged in an ambitious study in which they derived separate MDS solutions for the objects in each of the 240 categories from the Massive Memory database. To derive the individual-category MDS solutions, Hout et al. collected similarity data using an efficient method known as the spatial arrangement method (SpAM; Goldstone, 1994). In SpAM, observers are presented with a set of images and are tasked with arranging the images on a two-dimensional plane such that the distance between each pair of objects on the plane reflects their perceived dissimilarity. Because the complete set of similarities between objects composed of multiple dimensions cannot be captured by embedding them in a two-dimensional plane, the idea is to collect the spatial arrangements from multiple observers and to define the psychological dissimilarity between any given pair of objects as the average distance across the multiple spatial arrangements. A variety of MDS techniques can then be applied for modeling these derived psychological dissimilarities to achieve the goal of embedding the items in higher-dimensional similarity spaces. Hout and his colleagues have provided extensive evidence of the effectiveness of the SpAM approach for deriving MDS solutions in a wide variety of stimulus domains (e.g., Guevara Pinto et al., 2020; Hout & Goldinger, 2015; Hout et al., 2013; White et al., 2024).

Although the SpAM approach used by Hout et al. (2014) provides a highly effective procedure for measuring the relative similarity between pairs of items *within* each of the categories of the Massive Memory database, it is unclear whether their procedure provides high-fidelity information regarding the relative magnitude of those within-category similarities *across* the different categories. For example, it is unclear whether the procedure preserves information, say, that two particular apples are more similar to one another than are two particular backpacks. The reason is that, in constructing the two-dimensional spatial arrangements on each trial, participants are viewing a single category of objects one at a time. Under these conditions, it seems likely that participants will calibrate their placements of the individual items with respect to each specific category, without necessarily maintaining a common judgment scale across the complete set of 240 categories. For example, if Category A has items that are all highly similar to one another, it may still be the case that participants will place the two most dissimilar objects in the category quite far apart on the two-dimensional plane, because the participants’ main task is to preserve the relative dissimilarities of all the pairs of items *within* the category, not to judge their absolute dissimilarities across the categories.

Indeed, this assumption that participants will calibrate perceptual-magnitude ratings with respect to particular sets of objects being judged dates back to some of the earliest major theories of perceptual judgment. For example, in introducing his range-frequency model of perceptual-magnitude judgments for sizes of a set of squares, Parducci (1965) wrote:

‘Large’, like ‘beautiful’ or ‘worthwhile’, relates what is being judged to some implicit frame of reference. For the squares, the judgments adjust to the range of sizes in the experiment. After repeated presentations, ‘very large’ is reserved for the largest, ‘very small’ for the smallest, with the other [rating] categories indicating the order of the intermediate sizes. (p. 407)

Although Parducci’s argument is concerned specifically with judgments of the largeness of a set of squares, the same ideas translate directly to cases involving how observers calibrate their pairwise similarity judgments.

Because our current project on computational modeling of recognition memory demands that psychological distances between pairs of items be accurately measured *across* categories, we were thus led to explore an alternative approach to collecting the similarity-judgment data and deriving the associated MDS solutions. To be clear, with our proposed alternative method, we had no hope here of matching the efforts from Hout et al. (2014) of deriving MDS solutions for each of the 240 categories. Instead, given our goals, we limited our consideration to a randomly selected subset of 36 of the categories, with 13 items randomly sampled from each category.

Our alternative method involved several changes from the SpAM approach that had been used by Hout et al. (2014). First, rather than using SpAM, we used the more traditional method of asking for individual-trial direct judgments of similarity between pairs of items on a Likert scale. This procedure is admittedly far less efficient than is SpAM, but something like it may be needed to achieve our goal (see discussion below). The second modification was that, rather than collecting similarity judgments between items one category at a time, we randomly sampled on each trial which of the 36 categories the items were drawn from. So, for example, on trial *n*, the subject might provide a similarity judgment between two randomly selected apples, whereas on trial *n*+1 they might provide a similarity judgment between two randomly selected backpacks. Our thinking was that this random alternation procedure might allow subjects to maintain a more nearly constant judgment scale across the multiple categories, rather than recalibrating their judgment scale separately for each individual category. If so, then the similarity judgments both within and *across* the multiple categories could be meaningfully compared. In addition, as explained in the Modeling section of our article, we used a form of metric MDS modeling that would yield MDS solutions for the objects that would preserve information about overall differences in absolute similarity across the different categories. We will provide some preliminary evidence that this alternating-category rating procedure together with the metric form of MDS modeling may have been effective in achieving our goal.

Finally, before proceeding, we should explain why we chose not to use a method in which both within- *and* between-category judgments were collected across trials. For the present database of highly diverse categories, within-category similarities (e.g., similarities between different apples) are far greater than are between-category similarities (e.g, similarities between apples and backpacks). Besides the fact that there exists an enormous number of individual-item between-category pairs, making the collection of such data prohibitive, mixing between-category pairs with within-category ones would almost certainly reduce the fidelity of the within-category similarity judgments. If pairs of items were selected randomly across trials, then the overwhelming majority of pairs would be between-category pairs, and participants would likely adopt a scale of judgment appropriate to those between-category pairs. So, for example, the similarity judgment between an item from the category *breadloaf* and an item from the category *donut* would be extremely high compared to the judgment between an item from the category *breadloaf* and an item from the category *lamps*. (We provide documentation of this prediction later in our article.) Thus, on the small proportion of trials in which items from within the same category were judged, all judgments would likely be extremely high, and there would be a loss of fidelity in measuring the variations in degrees of within-category similarity among the items in the database. For our present project, it is the measurement of the within-category similarities that is crucial, which is why we decided to investigate the present alternating within-category similarity-judgment procedure.

## Alternating within-category similarity judgment experiment

### Method

#### Participants

The participants were 306 undergraduates from Indiana University Bloomington who took part in partial fulfillment of an introductory psychology course requirement. All participants had normal or corrected-to-normal vision, and all claimed to have normal color vision. The study was approved by the Institutional Review Board of Indiana University Bloomington.

#### Stimulus materials

The stimuli were images sampled from the “Massive Memory” Object Categories database of Talia Konkle (Konkle et al., 2010). We randomly sampled 36 of the 240 categories, and randomly sampled 13 of the exemplars from each category for use in the study, for a total of 468 images. The 36 categories are listed in Table 1. A complete listing of the 468 image filenames is provided in the Supplement (https://osf.io/aqb6w/).

**Table 1:** Weighted mean and standard deviation of within-category similarity judgments across the 78 cells of each of the 36 category matrices in both the simultaneous and sequential conditions

Category		Simultaneous		Sequential	
#	Name	Mean	StDev	Mean	StDev
1	Apple	5.636	1.28	5.641	1.66
2	Backpack	5.883	1.18	5.682	1.53
3	Ball	4.578	1.12	4.217	1.64
4	Bread	4.825	1.19	4.952	1.34
5	Butterfly	4.968	0.85	4.906	1.07
6	Carfront	3.718	1.31	4.136	1.57
7	Cat	4.800	1.20	4.617	1.34
8	Coffeemug	4.029	0.88	4.332	1.11
9	Cookpot	5.020	0.80	4.996	1.02
10	Desk	4.238	1.04	4.459	1.27
11	Donut	4.773	1.00	4.880	1.29
12	Earrings	3.618	0.86	3.929	1.19
13	Exercise	4.699	1.75	4.931	1.91
14	Garbagetrash	4.234	1.08	4.308	1.18
15	Glasses	4.762	1.06	5.157	1.29
16	Greenplant	4.827	0.83	5.224	1.16
17	Guitar	4.945	1.16	4.986	1.34
18	Hanger	4.518	1.07	4.754	1.25
19	Hat	4.017	1.04	4.394	1.21
20	Kettle	5.187	1.09	5.077	1.32
21	Key	4.007	1.25	4.568	1.38
22	Lamp	3.556	0.72	3.743	1.06
23	Leaves	3.897	1.14	4.200	1.28
24	Motorcycle	5.778	0.84	5.343	1.31
25	Paintbrush	5.464	0.95	5.429	1.17
26	Pants	5.094	1.25	4.894	1.41
27	Patiochair	4.660	0.88	4.899	1.18
28	Perfume	4.201	1.36	4.228	1.48
29	Powerstrip	5.197	0.93	5.324	1.24
30	Rock	3.961	1.16	4.029	1.28
31	Scissors	5.672	1.02	5.958	1.35
32	Shoe	4.028	1.16	4.153	1.26
33	Sink	4.871	1.26	5.011	1.44
34	Sofa	4.451	0.86	4.574	1.26
35	Stapler	4.596	1.12	4.752	1.61
36	Umbrella	5.575	1.02	5.442	1.29

The stimuli were presented on a 23-in. LCD computer screen. The stimuli were displayed on a white background. The size of the images varied slightly across categories; on average, they were approximately 1.57 × 1.57 in. Subjects sat approximately 20 in. from the computer screen, so on average each image subtended a visual angle of approximately 4.5° × 4.5°. The members of the pair of stimuli presented on each trial were horizontally centered around the central location on the screen and were separated by approximately 3.5 in. The experiment was programmed in MATLAB and the Psychophysics Toolbox (Brainard, 1997).

#### Procedure

On each trial of the similarity-judgment experiment, one of the 36 categories was randomly sampled. Next, two distinct images were randomly sampled from that category. The left–right placement of the members of the pair was randomized. The subject rated the similarity between the images on a scale from 1 (most dissimilar) to 9 (most similar). A visual depiction of the scale was provided on the bottom of the screen on each trial to remind the subjects of the rating system. Subjects were instructed that the images they would be viewing belonged to categories and that on all trials they would be comparing objects belonging to the same category. For example, on one trial they might be comparing two butterflies and on another trial two cars. It was explained to subjects that even within categories, some pairs of objects may be far more similar than other pairs and that they should try to use the full rating scale from 1–9, while keeping in mind that all pairs that they judged would be same-category pairs.^1^ No instructions were provided regarding the basis for the similarity judgments. Subjects based their judgments on whatever aspects of the images they deemed appropriate.

Each subject provided 360 judgments. We computed across all subjects the average similarity ratings for the 360 pairs of objects that a given subject had been presented with. We then computed the correlation between the individual subject’s 360 ratings and these averaged 360 ratings. Visual inspection of a histogram of the computed correlations indicated that subjects whose correlation with the average ratings was less than .40 were outliers, so we deleted these subjects from our subsequent analyses. Our thinking here was that such outliers were likely to add unwanted noise to the similarity measurements. Among the 306 subjects who were tested, this led to the deletion of 33 subjects (10.7%), leaving 273 for analysis. Because each of the 273 subjects provided 360 ratings, there was a total of 98,280 rating trials. There are 78 distinct pairs of the 13 stimuli in each of the 36 category-based similarity-judgment matrices, for a total of 2,808 distinct-pair cells across the 36 matrices. Thus, each cell had an average of 35 observations. Across the 2,808 cells, the minimum number of observations was 12 and the maximum was 60.

## Results and formal modeling analyses

**Fig. 1:**
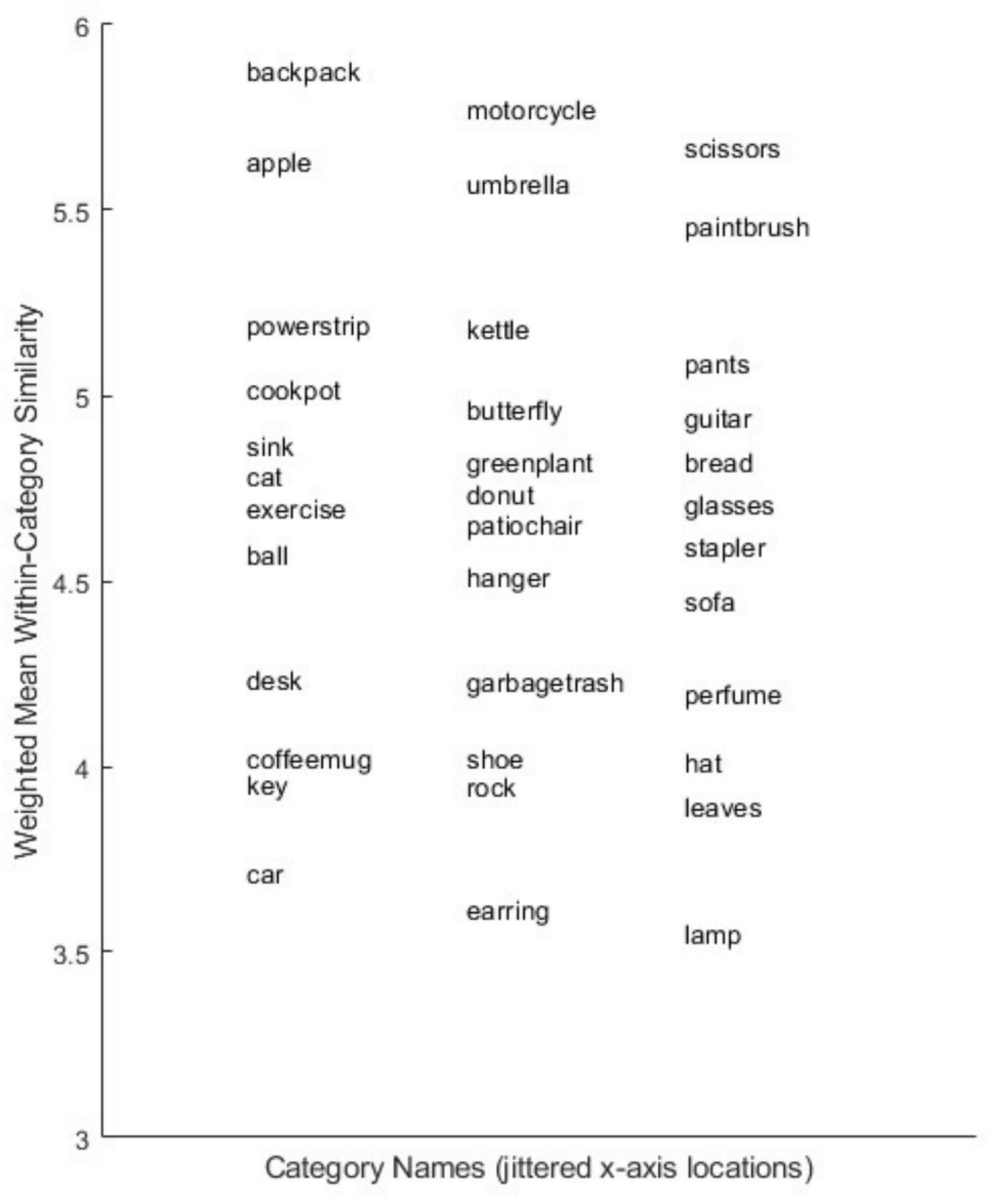
Jitter plot of weighted mean within-category similarity judgments

The individual-subject/individual-trial data from the experiment are available in the Supplement (https://osf.io/aqb6w/). The mean similarity ratings for each pair of items from each of the 36 categories, as well as the frequency of judgments for each pair, are also provided in the Supplement. In Table 1 (simultaneous condition), we report the frequency-weighted mean and standard deviation of the pairwise similarity ratings of each of the 36 categories. The mean category-level similarity judgments are also depicted in Fig. 1. A one-way repeated-measures analysis of variance (ANOVA) with Greenhouse–Geiser correction revealed a significant effect of category on the mean judgments, *F*(24.3, 6,637.7) = 130.6, mean squared error (*MSE*) = 1.254, *p* < .001. (Using JASP, a Bayesian ANOVA reported the Bayes factor in favor of the unequal-means model as *BF* = ∞.) Among the categories with high mean similarity ratings were backpack, motorcycle, scissors, and apple. Among the categories with low ratings were lamp, earring, and car-front.

Unlike in approaches in which MDS solutions are derived completely separately for each individual similarity-judgment matrix, the idea here was to derive the MDS solutions for each category by imposing certain constraints across the 36 matrices and to use metric forms of MDS modeling. For categories as diverse as these, we assumed that the objects in each category were characterized by different sets of dimensions (but see Hebart et al., 2020, for an approach in which a common high-dimensional space is derived for highly diverse categories). Thus, as in standard approaches, each item in each category was represented as a point in a *separate M*-dimensional Euclidean space. The coordinates of the points are free parameters in the model (although, as explained in the Appendix, various coordinate parameters can be held fixed at default values without loss of generality). The joint modeling procedure was as follows.

Let *d*_*k,ij*_ denote the distance between items *i* and *j* in the category-*k* MDS space. The predicted similarity rating for item-pair *i–j* in category *k* (*s*_*k,ij*_) was assumed to be a decreasing function of this distance. In a linear model, we assumed that

1 $${s}_{k,ij}=b-\nu {d}_{k,ij}$$

where *b* and *v* are freely estimated parameters. Crucially, the values of *b* and *v* are held fixed in generating the predictions across all 36 category matrices. The importance of this constraint is explained below.

Preliminary inspection of the modeling results suggested occasional curvature in the plots of measured similarity against distance in the derived MDS solutions. Therefore, we also investigated an exponential model, which assumed that

2 $$s_{k,ij}=b+u\cdot\exp\left(-vd_{k,ij}\right)$$

where *b*, *v*, and *u* are freely estimated parameters that are held fixed across all 36 category matrices.^2^

We conducted computer searches (see the Appendix for details) for the values of the 36 sets of MDS coordinate parameters and the fixed values of *b*, *v*, and *u* that minimized the frequency-weighted sum of squared deviations (wSSD) between the observed and predicted mean similarity ratings across all 78 cells of all 36 categories (2,808 mean similarity judgments). In the present metric MDS modeling approach, because the parameters *b*, *v*, and *u* are held fixed across all categories, and we are minimizing the wSSD across all 2,808 cells of the 36 matrices simultaneously, the “scales” of the different category MDS solutions are meaningfully compared. In categories in which items are judged as relatively dissimilar to one another, the points in the individual-category MDS solution will be more spread out than in categories in which items are judged as relatively similar to one another.

This approach differs somewhat from more typical “nonmetric” MDS approaches. In the nonmetric approaches, one searches for a configuration of points that minimizes *stress* (Kruskal & Wish, 1978), a measure of the extent to which the distances between points in the MDS solution are monotonic with the observed similarity data. Because the target goal is to model only the ordering of the similarities within each individual matrix, the overall “scale” of each category’s MDS solution is arbitrary—the points in each configuration can be multiplied by an arbitrary constant without changing the stress of that category’s MDS solution. Nevertheless, once a configuration with minimum stress is derived, nonmetric routines such as MATLAB’s MDSCALE will often multiply all the coordinates in the minimum-stress configuration by a scaling constant. The scaling constant is often chosen to minimize the SSD between the pairwise distances in the minimum-stress configuration and the measured empirical dissimilarities. Thus, the traditional nonmetric approach will tend to yield at least somewhat comparable results to the method we use here. The key difference is that minimizing SSD simultaneously across all 36 matrices is the target goal of the present metric approach, whereas minimizing stress within each of the individual matrices is the target goal of the nonmetric approach.

For simplicity, we considered only versions of the model in which all MDS solutions had the same dimensionality, and we varied the dimensionality from 2 through 5. Unfortunately, especially in cases involving complex, naturalistic stimuli, there is no consensus in the field for a “correct” method for selecting the appropriate dimensionality of MDS solutions. As dimensionality increases, the absolute fit of the MDS model will improve, but at the expense of incorporating numerous additional free coordinate parameters in the model. An early approach, suggested by Lee (2001), was to use model evaluation statistics that penalize the fit of the MDS model based on its number of free parameters. For completeness, we report results from such analyses in Table 2, using both the Akaike information criterion (AIC; Akaike, 2003) and Bayesian information criterion (BIC; Schwarz, 1978) statistics—see the Appendix for the details of this approach. Smaller values of AIC or BIC point to the preferred model. The AIC tends to favor more complex models with more free parameters, whereas the BIC tends to favor simpler models with fewer free parameters. As shown in Table 2, regardless of dimensionality, both the AIC and BIC statistics strongly favor the exponential model over the linear one. However, the AIC favors a four-dimensional MDS model, whereas the BIC favors a two-dimensional one. Naturally, the frequency-weighted proportion of variance accounted for across the 2,808 individual cells increases with increasing dimensionality (see Table 2). In addition, at the aggregated category level (see Table 1), all models accounted for over 0.98 proportion of the variance.

**Table 2:** Summary fit statistics for the alternative multidimensional scaling models

Linear model							
*N*_dim_	C/matrix	P Tot	wSSD	wMSD	BIC	AIC	w%Var
2	23	829	949.4	0.338	16,482.3	11,557.9	78.3
3	33	1,189	560.4	0.200	15,284.5	8,221.6	87.2
4	42	1,513	339.0	0.121	15,548.5	6,560.9	92.2
5	50	1,801	211.3	0.075	16,503.7	5,805.3	95.2
Exponential model							
*N*_dim_	C/matrix	P Tot	wSSD	wMSD	BIC	AIC	w%Var
2	23	830	630.2	0.224	**13,161.8**	8,231.4	85.6
3	33	1,190	362.1	0.129	13,224.7	6,155.8	91.7
4	42	1,514	232.1	0.083	14,441.7	**5,448.2**	94.7
5	50	1,802	183.9	0.065	16,225.9	5,521.6	95.8

Note. *N*_dim_ = number of dimensions; C/matrix = no. of free coordinate parameters per matrix; P Tot = total number of free parameters for the MDS model across the 36 matrices; wSSD = weighted sum of squared deviations between predicted and observed individual-cell similarity ratings; wMSD = weighted mean squared deviation between predicted and observed individual-cell similarity ratings; BIC = Bayesian information criterion; AIC = Akaike information criterion; w%Var = weighted percentage of variance accounted for in the individual-cell similarity ratings across the 36 matrices

Gronau and Lee (2020) provide discussion that strongly questions the use of AIC and BIC statistics for selecting the appropriate dimensionality of MDS solutions, because the manner in which the very large number of coordinate parameters may interact and constrain each other is unknown. As an alternative, they initiated the investigation of a Bayesian inference approach to choosing the appropriate number of dimensions. However, the Bayesian inference approach faces technical challenges, and Gronau and Lee (2020, p. 336) report that the approach cannot currently be applied in cases involving large-size naturalistic stimulus domains.

Steyvers (2002) provided a brief investigation into the use of cross-validation for choosing MDS dimensionality; to our knowledge, however, the approach has not taken hold in the field. In exploratory fashion, we decided to pursue that approach here, although the extent to which the approach leads to robust conclusions is unknown.

In one approach, we divided the full data into two halves, with all odd-numbered participants composing a “training” data set and all even-numbered subjects composing a “test validation” set. Using the wSSD measure, we fitted both the exponential and linear MDS models to the individual-cell training-set data at each of dimensionalities 2–5. Then, holding fixed all the best-fitting MDS model parameters, we computed the resulting individual-cell test set wSSD values. To eliminate the influence of the total number of observations across the training and test sets on the wSSD measure, we then computed the weighted mean squared deviation (wMSD) in each case. The results are reported in Table 3. Naturally, the fits to the training-set data improve as the dimensionality of the MDS solution increases. In addition, as expected, the fits to the test-set data are worse than to the training-set data, because the parameters were not tuned to the test-set data. Nevertheless, the test-set fits are consistently better for the exponential model than for the linear one, in agreement with the pattern of AIC and BIC results reported in Table 2. The best test-set validation fit is for the 4D exponential model, although the improvement compared to the 3D and 5D exponential models is small in magnitude. We conducted a second set of analyses using fivefold cross-validation rather than split-half cross-validation; the qualitative pattern of results was similar to the one that we have already reported.

**Table 3:** Cross-validation analyses for MDS models with dimensionality varying from 2 to 5

Weighted mean squared deviations between observed and predicted individual-cell similarity ratings				
	Exponential model		Linear model	
ndim	Training	Test	Training	Test
2	0.2758	0.4662	0.3910	0.5617
3	0.1708	0.4101	0.2374	0.4740
4	0.1121	0.3928	0.1491	0.4382
5	0.0813	0.3969	0.0968	0.4187

In a nutshell, overall, although the results favor the exponential model over the linear one, they did not leave us with very strong motivation for focusing on one dimensionality versus the others. We report the best-fitting MDS solutions at each dimensionality in our Supplement. In the following sections of the article, we consider in greater depth the results from the 3D and 4D models.

In Table 4 we report for both the 3D and 4D linear and exponential models the weighted proportion of variance accounted for in the individual cells broken down by the individual categories. Although there are occasional exceptions (e.g., the earring and lamp categories), in general the proportion of variance accounted for by the models seems reasonably high. Across the 36 categories, the 3D linear model accounts for an average of 0.804 of the variance and the 3D exponential model for an average of 0.875 of the variance; the 3D exponential model yields a better fit than the 3D linear model for 32 of the 36 categories. The 4D linear model accounts for an average of .880 of the variance and the 4D exponential model for an average of .921 of the variance; the 4D exponential model yields a better fit than the 4D linear model for 31 of the 36 categories.

**Table 4:** Weighted proportion of variance accounted for by the 3D and 4D linear and exponential models for the individual cells in each individual category matrix

		3D MDS model		4D MDS model	
Cat #	Cat name	Linear	Expon.	Linear	Expon.
1	Apple	0.840	0.935	0.905	0.967
2	Backpack	0.891	0.934	0.938	0.963
3	Ball	0.879	0.921	0.925	0.953
4	Bread	0.894	0.883	0.939	0.931
5	Butterfly	0.819	0.851	0.874	0.895
6	Car	0.840	0.902	0.939	0.935
7	Cat	0.797	0.886	0.883	0.943
8	Coffeemug	0.651	0.824	0.816	0.908
9	Cookpot	0.777	0.725	0.855	0.860
10	Desk	0.859	0.912	0.912	0.949
11	Donut	0.826	0.839	0.887	0.893
12	Earring	0.574	0.836	0.723	0.896
13	Exercise	0.942	0.968	0.966	0.983
14	Garbage/Trash	0.825	0.844	0.884	0.912
15	Glasses	0.838	0.917	0.917	0.957
16	Greenplant	0.736	0.809	0.872	0.888
17	Guitar	0.919	0.919	0.952	0.937
18	Hanger	0.844	0.909	0.880	0.932
19	Hat	0.769	0.909	0.853	0.954
20	Kettle	0.921	0.953	0.959	0.949
21	Key	0.867	0.944	0.927	0.964
22	Lamp	0.174	0.770	0.453	0.835
23	Leaves	0.871	0.849	0.926	0.893
24	Motorcycle	0.770	0.794	0.862	0.898
25	Paintbrush	0.829	0.863	0.885	0.905
26	Pants	0.855	0.893	0.904	0.931
27	Patiochair	0.813	0.855	0.906	0.914
28	Perfume	0.801	0.905	0.883	0.942
29	Powerstrip	0.804	0.842	0.881	0.898
30	Rock	0.771	0.869	0.851	0.877
31	Scissors	0.884	0.921	0.944	0.940
32	Shoe	0.875	0.919	0.931	0.945
33	Sink	0.820	0.850	0.873	0.894
34	Sofa	0.666	0.774	0.772	0.834
35	Stapler	0.848	0.911	0.895	0.933
36	Umbrella	0.839	0.873	0.907	0.932
	Avg.	0.804	0.875	0.880	0.921

**Fig. 2:**
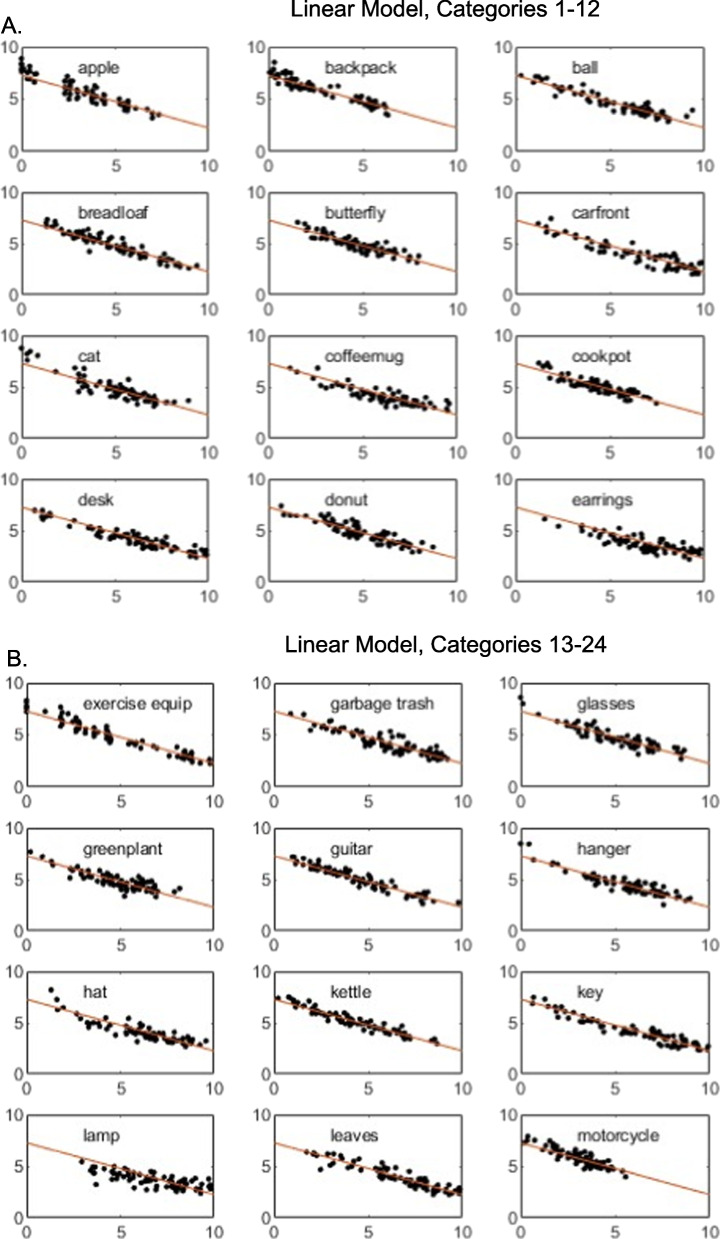

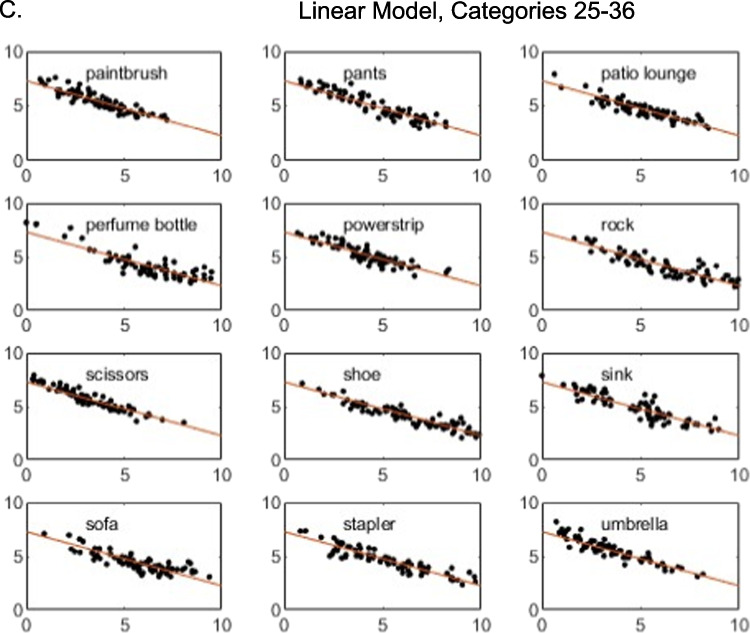
**A** Weighted mean similarity judgments (*y*-axis) plotted against MDS-derived distances (*x*-axis), categories 1–12. Three-dimensional linear model. **B** Weighted mean similarity judgments (*y*-axis) plotted against MDS-derived distances (*x*-axis), categories 13–24. Three-dimensional linear model. **C** Weighted mean similarity judgments (y-axis) plotted against MDS-derived distances (*x*-axis), categories 25–36. Three-dimensional linear model

**Fig. 3:**
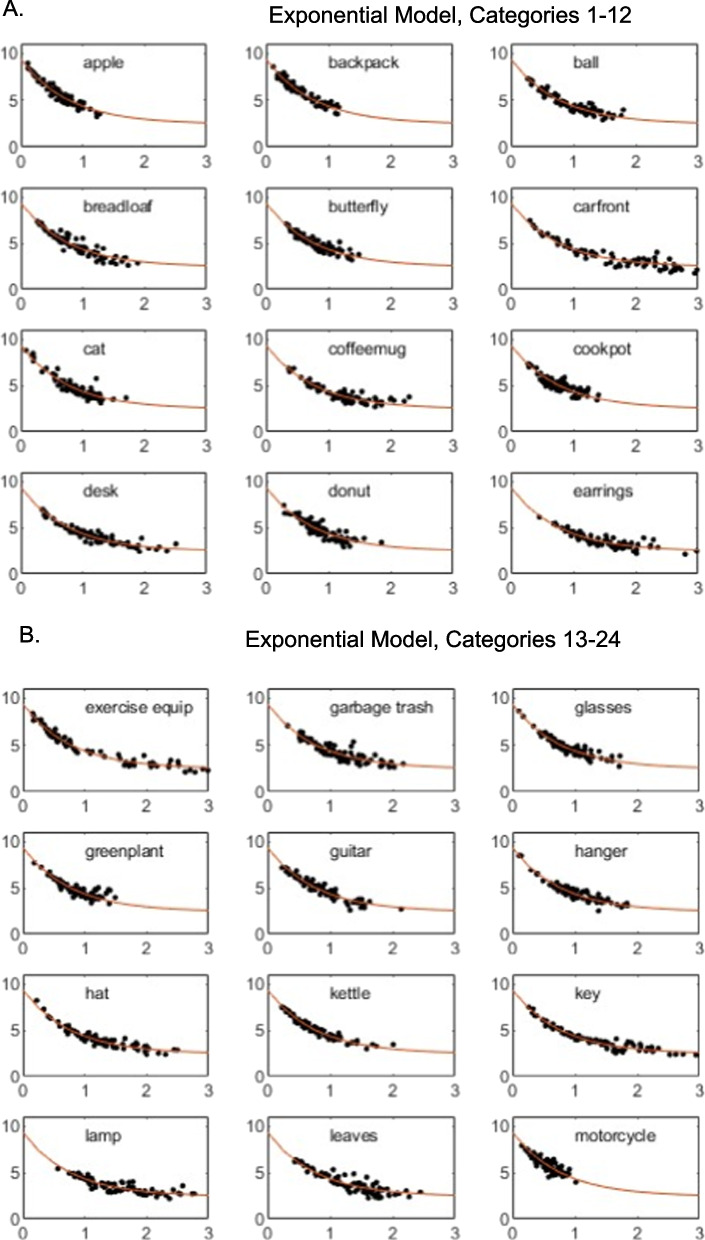

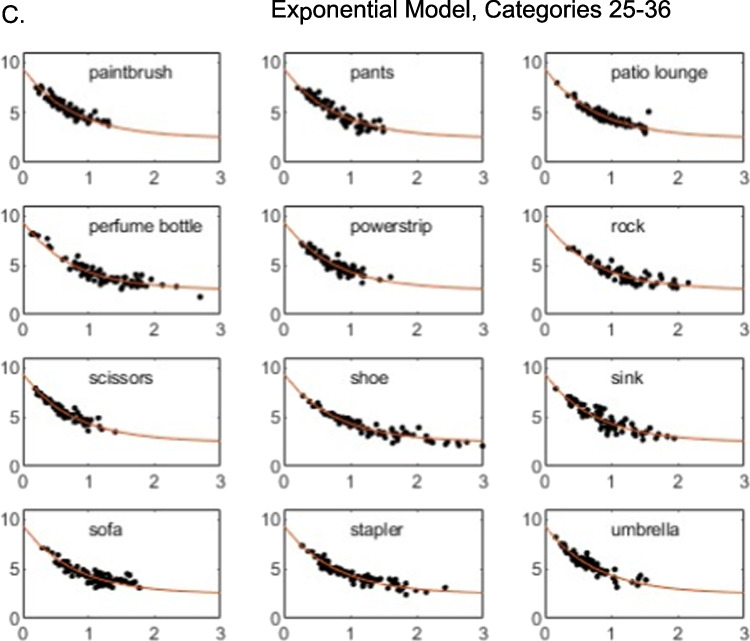
**A** Weighted mean similarity judgments (*y*-axis) plotted against MDS-derived distances (*x*-axis), categories 1–12. Three-dimensional exponential model. **B** Weighted mean similarity judgments (*y*-axis) plotted against MDS-derived distances (*x*-axis), categories 13–24. Three-dimensional exponential model. **C** Weighted mean similarity judgments (*y*-axis) plotted against MDS-derived distances (*x*-axis), categories 25–36. Three-dimensional exponential model

To provide a deeper perspective on the model fits, in Figs. 2 and 3, for each individual category we present plots of the observed individual-cell mean similarity judgments against distances in the derived 3D MDS solutions, along with the theoretical function relating similarity to distance in the space. Inspection of the figures reveals that the high proportion of variance accounted for by the models does not arise simply because some pairs have very high similarity and others very low similarity. Instead, similarity varies in continuous fashion as a function of distance in the space, and the observed data follow the prediction curves fairly well. The plots reveal, however, some systematic departures of the data from the predictions of the linear model: in numerous cases in which some individual pairs in the categories yielded high similarity ratings, those ratings are under-predicted by the linear model. By comparison, the exponential model does a good job of capturing those cases. Although past work has often assumed the linear model when deriving metric MDS solutions from similarity-ratings data (e.g., Nosofsky et al., 2011, 2018), the present results suggest that nonlinear models such as the exponential may be more appropriate.

### Toward validation of the derived MDS solutions

As one approach to illustrating the potential utility of the derived MDS solutions, in this section we present preliminary evidence that the solutions are useful for predicting performance in an independent cognitive task. We are currently engaged in a project aimed at developing cognitive-process models of old–new recognition of images of real-world visual objects (Osth & Nosofsky, in preparation). In a separate report, we plan to present data and formal modeling analyses from a series of old–new recognition experiments involving the present stimuli. In one of these experiments, 226 participants studied a list of 234 of the 486 images from the present MDS project. The list was composed of six or seven randomly selected objects from each of the 36 categories. Following this study phase, subjects were presented with a test list composed of all 486 images and were required to judge whether each item was old or new. All subjects saw the same set of study and test items, but the order of presentation of the items was fully randomized for each individual subject.

A simple baseline model of individual-item old–new recognition is Nosofsky’s (1988; Meagher & Nosofsky, 2023) *generalized context model* (GCM). According to the model, the “familiarity” of test item *i* (*F*_*i*_) is found by summing its similarity to all items on the study list. The similarity of item *i* to each study item *j* (*s*_*ij*_) is assumed to be an exponentially decreasing function of the distance (*d*_*ij*_) between *i* and *j* in the derived MDS solution (Shepard, 1987; see also Marjieh et al., 2024),

3 $${s}_{ij}=\mathrm{exp}\left(-v{ d}_{ij}\right)$$

where *v* is a freely estimated overall sensitivity parameter. In the present application, these interitem similarities are computed only among items *within* the same categories, because separate MDS solutions were derived for the separate categories. For simplicity, we estimate a small residual *between*-category summed-similarity value (*B*) between all items belonging to different categories. The overall familiarity for any given test item *i* belonging to some category *C* is given by

4 $${F}_{i}={\left[\sum_{j\varepsilon C}{s}_{ij}+B\right]}^{\gamma }$$

where the power-parameter γ makes allowance for nonlinear relations between psychological familiarity and summed similarity.

The probability with which item *i* is judged to be “old” is then given by

5 $$P\left(\left.\mathrm{Old}\right|i\right)={F}_{i}/\left({F}_{i}+k\right)$$

where *k* is a criterion parameter. Test items that give rise to *F*_*i*_ values greater than *k* tend to be called old, whereas test items that give rise to *F*_*i*_ values less than *k* tend to be called new. The model estimates only four free parameters for fitting the test-trials data: *v*, *B*, γ, and *k*. For simplicity, in the present illustrative application we held these parameters fixed across the 226 participants and used a computer search to find the values that provided a maximum-likelihood fit to the individual-trial old–new recognition data. In particular, let *p*_*n*_ denote the predicted probability of an “old” response on some individual trial *n*, and let δ_*n*_ be an indicator variable set equal to 1 if an old response was given on trial *n* and set equal to 0 otherwise. Then the criterion of fit was to find the free parameters that minimized the negative log-likelihood statistic

$$-\mathrm{ln}L=-\sum_{n}\left[{\delta }_{n}\mathrm{ln}\left({p}_{n}\right)+\left(1-{\delta }_{n}\right)\mathrm{ln}\left(1-{p}_{n}\right)\right]$$

We fitted the model to the individual-trial data using six different candidate sets of MDS solutions, namely the 3D and 4D solutions derived from (i) the exponential MDS model, (ii) the linear MDS model, and (iii) the nonmetric scaling solutions produced by MATLAB’s MDSCALE. (Note that all six versions of the recognition model use four free parameters, because the solutions are held fixed from what was derived by fitting the different MDS models to the similarity-ratings data.) The fits to the individual-trial old–new recognition data are reported in Table 5. As can be seen, the exponential model yields the best fits to the data.

**Table 5:** Summary fits of the baseline old–new recognition model to the individual-trial data and the category-level false-alarm-rate data using the alternative MDS solutions as a source of input

	Fit statistic	
MDS solution	Individual trials, -ln*L*	Cat.-level FA rate, *r*
3D Nonmetric	66,228.4	0.57
4D Nonmetric	66,156.7	0.60
3D Linear	66,241.3	0.68
4D Linear	65,986.6	0.69
3D Exponential	65,910.7	**0.70**
4D Exponential	**65,857.8**	0.69

Note. -ln*L* = negative log-likelihood; FA rate = false-alarm rate; *r* = Pearson correlation coefficient. Best fits are indicated in boldface font

**Fig. 4:**
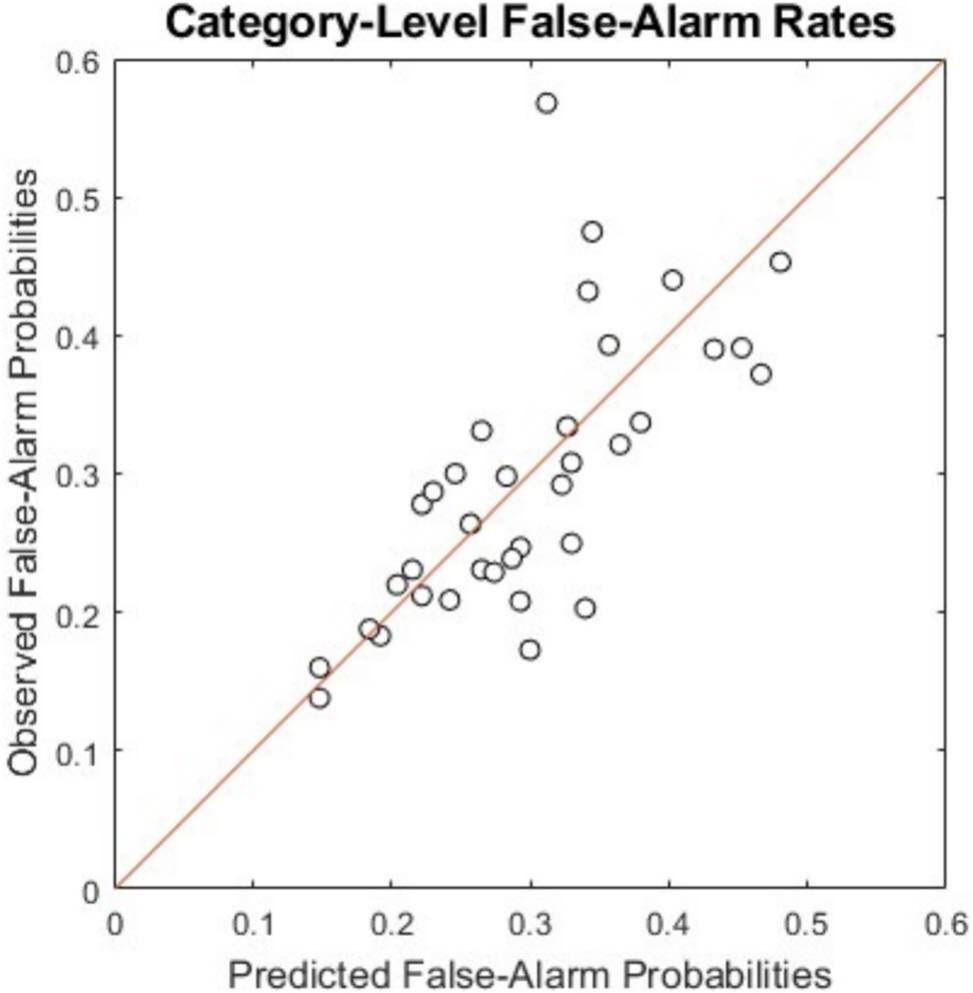
Observed against predicted category-level false-alarm rates in the old–new recognition experiment (Osth & Nosofsky, in preparation). Predictions are from a baseline summed-similarity model of recognition that uses the three-dimensional exponential MDS solution as a source of input

Given the maximum-likelihood parameters from the models, we then computed category-level observed and predicted false-alarm rates by aggregating the individual-trial data and predictions. For example, the mean observed false-alarm rate for the category of apples was computed by dividing the total number of trials in which participants judged that a new apple was old by the total number of trials in which a new apple was presented. In Fig. 4 we provide a summary plot of these observed against model-predicted category-level false-alarm rates for the 3D exponential model. Visual inspection reveals that, with the exception of a single outlier point in the scatterplot, this simple model yields reasonably good predictions of the observed category-level false-alarm-rate data. This result provides a preliminary source of validation that our proposed trial-by-trial alternating-category approach to collecting similarity-judgment data was effective in maintaining information about the relative discriminability of the items *across* the different categories and that this information could be used to predict performance in an independent cognitive task. We acknowledge, however, that the correlations between the predicted and observed category-level false-alarm-rate data were essentially the same for the 3D and 4D linear and exponential MDS solutions (see Table 5). The improved individual-trial summary fits from the exponential-MDS solution that we reported in Table 5 involve some subtle quantitative improvements to a variety of aspects of the recognition data rather than being due to any major qualitative differences in the patterns of predictions.

It is of interest to note that the outlier point in Fig. 4 corresponds to the exercise equipment category. Inspection of Table 1 reveals that this category exhibited the greatest standard deviation of similarity judgments among the 36 categories. The large standard deviation reflects the fact that the category was composed of two subcategories: stationary bicycles and treadmills. Within-subtype similarity judgments in this category were relatively high, and between-subtype similarity judgments were lower. None of the other categories had an analogous breakdown into two distinct subtypes. Future research is needed that takes better account of the role of subcategories in mediating both the similarity and old–new recognition judgments. In any case, in our view, in an overall sense Fig. 4 displays promising results that provide evidence of the utility of the derived MDS solutions for capturing similarity relations among the objects both within and across these everyday-object categories.

## Supplementary ratings data for the objects in the categories

To supplement the within-category similarity-ratings data reported in our main experiment above, we collected several other forms of ratings data. We briefly describe each of these supplementary data sets below. In all cases, the stimuli were the same 468 images used in the main experiment, and the same apparatus was used.

### Similarity judgments for sequentially presented items

We conducted an exploratory follow-up of the main similarity-judgment experiment. The procedure for the follow-up experiment was the same in all respects as the main experiment, except that instead of presenting each pair of items simultaneously on each trial, the items were presented in sequential fashion, with a 1.5-s blank-screen interval between the presentation of the first item in the pair and the second item. This follow-up was motivated in part by the outlier false-alarm rate for the exercise equipment category that we discussed in the previous section. In addition to being composed of two subtypes, the objects in the exercise category were composed of multiple complex parts that were likely difficult to hold in memory in the context of an old–new recognition experiment. By comparison, in the main similarity-judgment experiment, each pair of objects was viewed simultaneously, and observers did not need to hold the objects in memory when making their judgments. By using a sequential-presentation procedure in our follow-up experiment, we thought that we might come closer to measuring the type of memory-based similarity more relevant for an old–new recognition task. We hypothesized that mean similarity judgments for the exercise category might increase differentially (relative to the other categories) in the sequential-presentation experiment compared to the simultaneous-presentation one.

### Method

#### Participants

The participants were 51 undergraduates from Indiana University who took part in partial fulfillment of an introductory psychology course requirement.

#### Procedure

The procedure was the same as in the main similarity-judgment experiment, except that the items were presented in sequential rather than simultaneous fashion. The first item in the pair was still presented on the left side of the screen (for 1.5 s), and the second item on the right, but there was a 1.5-s blank-screen interval between the presentation of the first and second items. The second item remained on the screen until the subject entered their similarity judgment. Because individual trials in the sequential version of the experiment took longer than in the simultaneous version, we reduced the number of trials from 360 to 300.

### Results

**Fig. 5:**
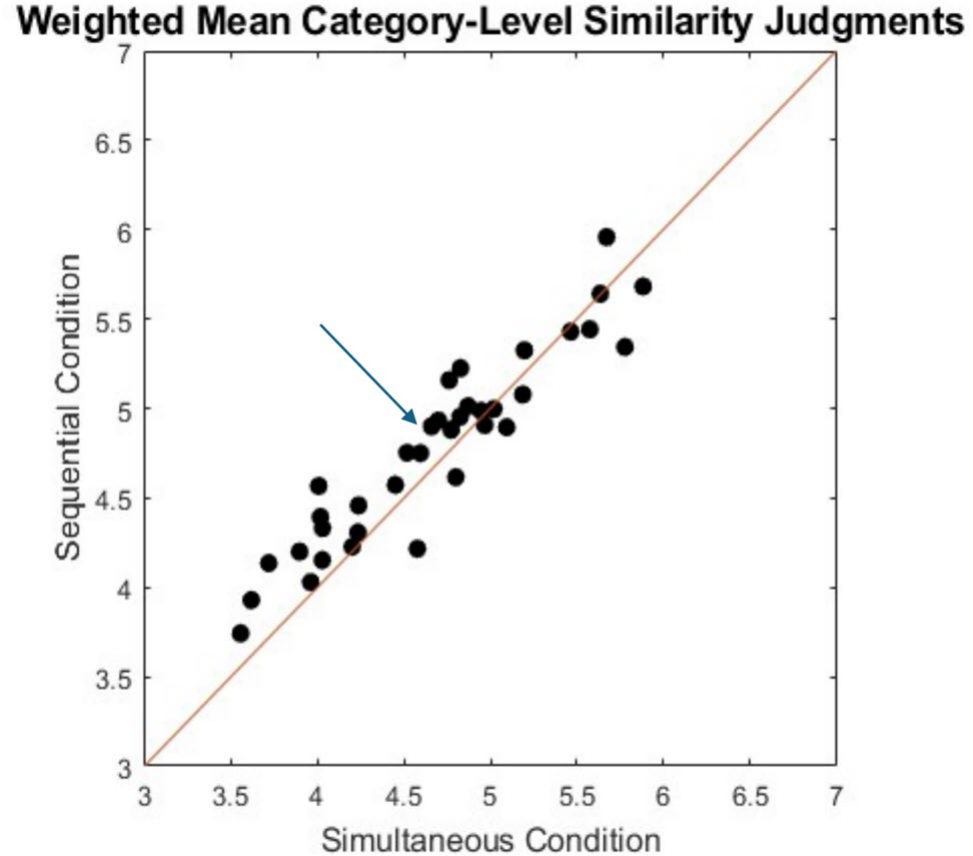
Weighted mean category-level similarity judgments in the sequential presentation condition plotted against weighted mean category-level similarity judgments in the simultaneous presentation condition. The arrow indicates the results for the exercise equipment category

The individual-subject trial-by-trial data files are available in the Supplement. There were insufficient data for a meaningful MDS analysis of the individual-item similarity judgments. Instead, our main interest was to explore whether the category-level judgments may have changed in systematic fashion across the simultaneous- and sequential-presentation studies. The mean frequency-weighted category-level similarity judgments from the sequential experiment are reported along with the simultaneous ones in Table 1. In Fig. 5 we display a scatterplot of the mean category-level similarity judgments observed in the sequential condition against those observed in the simultaneous condition. As can be seen, the category-level means were extremely similar across the two conditions, *r*(36) = 0.941. This result is important in and of itself because it provides additional evidence that our alternating-category similarity-rating procedure produces reliable results for measuring differences in the magnitude of within-category similarities across the different categories. Regarding the more nuanced question that partly motivated the experiment, however, we obtained little support for our hypothesis that the mean judgments for the exercise equipment category would increase differentially (compared to the other categories) in the sequential condition—see Fig. 5.

## Individual-item distinctive-feature ratings

We conducted a study in which participants provided “distinctive-feature” ratings for the 468 items. The general motivation was that computational cognitive models of old–new recognition make allowance for the idea that individual items may have differing degrees of “self-similarity” because of the presence of distinctive features (e.g., Meagher & Nosofsky, 2023; Nosofsky et al., 2025; Nosofsky & Zaki, 2003). For example, a face with a highly prominent scar may provide a better match to itself than does a face without such a distinctive feature (Tversky, 1977). The present data could therefore provide a potentially useful source of additional input for the application of such models to predicting performance in old–new recognition experiments.

### Method

#### Participants

The participants were 40 undergraduates from Indiana University who took part in partial fulfillment of an introductory psychology course requirement. All participants had normal or corrected-to-normal vision, and all claimed to have normal color vision.

#### Procedure

Subjects were instructed that they would be viewing images of objects from everyday categories. They were asked to rate the extent to which each object contained a highly distinctive feature that made it stand out from other objects in the set. They were given the example that if they were viewing faces, then a face with a highly prominent scar would have a highly distinctive feature. The subjects were instructed that a feature should be judged as “distinctive” only if it rarely occurs; if many objects contained the same feature, then it should not be rated as distinctive. Subjects provided ratings on a scale from 1 to 9, with 1 corresponding to low distinctiveness and 9 corresponding to high distinctiveness. Subjects were instructed that most of the objects that they would view did not contain distinctive features and that they should reserve high ratings for objects that contained truly distinctive features.

On each trial, the subject provided a rating for an image that was randomly selected (without replacement) from the complete set of 468 images. There were a total of 360 rating trials. Each image was rated by an average of 30.8 subjects.

### Results

The mean distinctive-feature ratings for the 468 individual images are reported in the Supplement along with the individual image filenames. In Table 6 we report the mean distinctive-feature ratings at the level of categories. Categories with relatively high mean ratings included butterfly, earring, and lamp. Categories with relatively low mean ratings included apple, garbage/trash, and paintbrush. To the extent that individual items within a category have different distinctive features, one might expect that they would tend to have lower similarity to one another relative to categories without items that have distinctive features. In agreement with this line of reasoning, the correlation between the mean category-level similarity ratings (Table 1, simultaneous condition) and the distinctive-feature ratings (Table 6) is *r*(34) = −0.41, *p* = .013. (Using JASP, the Bayes factor in favor of the nonzero correlation model was *BF* = 4.05.) Although the correlation is statistically significant, it is modest in magnitude, so the two types of ratings appear to provide partially independent components of information regarding the properties of the objects in the categories.

**Table 6:** Mean category-level distinctive-feature ratings

1	apple	1.84
2	backpack	2.42
3	ball	2.47
4	breadloaf	2.37
5	butterfly	5.03
6	carfront	4.32
7	cat	2.37
8	coffeemug	4.20
9	cookpot	2.59
10	desk	2.90
11	donut	2.64
12	earring	4.54
13	exercise	2.27
14	garbage	1.93
15	glasses	2.66
16	greenplant	2.40
17	guitar	3.84
18	hanger	2.72
19	hat	3.06
20	kettle	3.77
21	key	2.84
22	lamp	4.83
23	leaves	2.27
24	motorcycle	3.53
25	paintbrush	1.67
26	pants	2.16
27	patiochair	3.12
28	perfume	4.54
29	powerstrip	2.38
30	rock	3.31
31	scissors	2.81
32	shoe	2.52
33	sink	2.50
34	sofa	2.34
35	stapler	3.48
36	umbrella	2.46

### Discussion

#### Summary

In this article we proposed and implemented an approach to collecting similarity-judgment data from diverse everyday-object categories and conducting MDS modeling of those data. The goal was to collect data that provided good measurement of judged similarity for objects within categories and that also preserved information regarding the relative magnitude of those within-category similarities *across* the different categories. The key idea in the procedure was that, rather than collecting similarity-judgment data from the categories one at a time, we alternated randomly across trials the category from which the to-be-judged objects were selected. Our hope was that this procedure would allow participants to maintain a roughly constant judgment scale across the categories, rather than recalibrating their judgment scale for each individual category had the categories been judged one by one. We also implemented a metric multidimensional scaling procedure that enabled the derivation of MDS solutions for each category that would preserve any across-category differences in the overall magnitude of the judged within-category similarities. In other words, if the objects in Category A tended to be judged as more dissimilar to one another than the objects in Category B, then the points in the Category-A MDS solution would be more spread out than those in the Category-B MDS solution.

It is probably the case that no method for achieving the above-stated goals is perfect, and we describe below various likely limitations of the present proposed approach. Nevertheless, the preliminary evidence reported in our article suggests that the proposed approach was reasonably successful. First, inspection of the model-fit statistics in our Tables 2 and 4 reveals that the MDS models yielded good quantitative accounts of similarities within the individual categories as well as their relative magnitudes across the different categories. These good fits were not the result of simply capturing similarities of extremely high versus low magnitude. Instead, inspection of Figs. 2 and 3 reveals that judged similarities between pairs of items varied in continuous fashion within all of the categories and that the models provided good quantitative accounts of this continuous variation. It is also apparent from inspection of the figures that the models captured in quantitative detail the continuous variation in the overall magnitude of the similarities across the different categories.

Finally, we provided preliminary evidence of the utility of the approach by showing that the derived MDS solutions were useful for predicting performance in an independent cognitive task in which participants’ recognition memory for the objects from the diverse categories was tested. Here, the derived MDS solutions were used as input to a simple baseline model of old–new recognition memory. Given the input of the derived MDS solutions, the baseline model predicted reasonably well the variation in the false-alarm rates associated with the members of the different categories (Fig. 4). In future work, we plan more in-depth examinations of the extent to which models can quantitatively predict old–new recognition data involving objects from the present categories, but these preliminary results show promise that we are on the right path.

### Limitations

#### Limitations of the empirical program of research

In line with past historical arguments for related paradigms (e.g., Parducci, 1965), we argued that if the similarity-judgment data were collected one category at a time, participants would be likely to recalibrate their judgment scale with respect to each individual category being judged. If such individual-category recalibration of the judgment scale took place, it would reduce one’s ability to measure any true across-category differences in the magnitude of the psychological similarities. Admittedly, however, we did not provide an empirical demonstration that the one-category-at-a-time approach would indeed have provided less accurate measurement of the across-category similarities than our proposed alternating-category approach. The bottom line is that engaging in massive forms of data collection to verify the likely limitations of the one-category-at-a-time approach does not seem to us to be an efficient use of our limited research resources. Instead, we provided a positive demonstration of at least one data collection method that does seem highly promising for simultaneously measuring the relative magnitude of both within- and across-category similarities in cases involving highly diverse everyday-object categories.

#### Limitations of the proposed data collection method

A limitation of our proposed alternating-category method is that participants may have difficulty keeping track of previous judgments of pairwise similarity within the individual categories. Because the to-be-judged category is randomly selected on each trial, there will often be long lags between trials in which items from any given category are judged. Thus, memories for the previous judgments within a category may be weakened relative to paradigms in which each category is judged one at a time. Because judgments on a current trial are made relative to judgments made on previous trials, these weakened memories are likely to add noise to the within-category judgment process. Nevertheless, the promising results reported in this article suggest that the mix of judgments from other categories may provide a suitable general context for allowing the participants to maintain a consistent judgment scale across the trials of the experiment. Future research might investigate more sophisticated methods of interleaving across categories by using forms of adaptive-design optimization in which the specific pairs chosen for judgment on any given trial are those that maximally reduce uncertainty in the inferred MDS representations.

A second limitation is that our paradigm provided no information regarding between-category similarities. In the present domain, we hypothesize that the types of “similarities” that have a major impact on tasks such as old–new recognition memory are relatively small when considered at the between-category level compared to the within-category level. For example, although it is psychologically plausible that two apples may be confused in memory with one another, it seems unlikely that memory-based confusion would exist between an apple and a backpack. Nevertheless, certain types of between-category similarities may be relevant for other cognitive tasks, such as ones in which participants make analogical judgments (e.g., Ichien et al., 2023). In our Supplement, we present results from an experiment in which certain types of between-category similarity ratings were collected for the present stimuli. In brief, rather than making judgments of similarity between single items from contrasting categories (which would require an enormous number of individual-item between-category judgments), participants were instead presented with collections of items from contrasting categories and judged the similarity between the collections. Among the types of category pairs that received relatively high between-category ratings were cases involving common superordinate-category membership, such as types of food (apple-breadloaf), clothing (hat-pants), furniture (desk-sofa), and so forth. In other cases, the high similarities involved interactive relations between categories, such as butterfly and leaves, car-front and key, and lamp and powerstrip. The vast majority of the between-category similarity judgments were relatively low. It is an open question whether these different types of similarity may contribute to performance on the wide variety of cognitive tasks that might make use of the present stimuli.

A third limitation of our proposed method is that only a single direct judgment of pairwise similarity is collected on each trial. Potentially more efficient methods of data collection use paradigms in which multiple items are judged simultaneously (for a recent tutorial review of these and other methods, see Daggett & Hout, 2025). For example, in an odd-one-out method (e.g., Hebart et al., 2020; Romney et al., 1993), triplets of items are presented, and the participant chooses the item that is most dissimilar to the other two. In a choice paradigm used by Roads and Mozer (2019), a target item is presented along with comparison items, and observers choose in a sequence the comparison items that they judge to be most similar to the target. In identification-confusion paradigms, a single item from a set of *n* items is presented on each trial, and participants identify with a unique response which of the *n* items they judge had been presented (e.g., Nosofsky, 1985; Shepard, 1958; Townsend, 1971). Formal models are then used to account for the choice-probability data, allowing for the derivation of MDS solutions for the objects. It is an open question whether the use of these methods involving discrete choices of multiple options may provide more efficient and effective procedures than the use of continuous judgments involving single pairs. Future research will need to compare these alternative methods of deriving MDS solutions for objects from diverse, everyday object categories.

#### Limitations of the MDS approach

Finally, questions arise regarding the importance of the MDS modeling itself. In the present case, the MDS modeling served mainly as a data-reduction technique in which the pairwise similarity-judgment matrices were compressed, potentially reducing the noise in the raw similarity-judgment data. In many past applications, MDS modeling has played a more important psychological role, because it provides a foundation for the modeling of systematic changes in similarity relations across different task contexts. For example, in applying the GCM to predict categorization and old–new recognition, Nosofsky (1991) used dimension-weight parameters in concert with derived MDS representations. The weight parameters were intended to reflect psychological changes in attention to dimensions that were differentially relevant for performing the categorization and recognition tasks. Meaningful applications of this approach, however, typically require that the derived MDS dimensions have natural psychological interpretations. In the present case, we have not yet pursued the path of finding psychological interpretations for the dimensions of the individual-category MDS solutions. Pursing such a path is likely to increase the usefulness of the derived MDS representations as a source of input to computational models of cognitive processes.

Furthermore, alternative representational models might also be applied to the present sets of similarity-judgment matrices. For example, in recent work, Westfall and Lee (2021) successfully applied models based on discrete-feature matching to account for changes in the structure of semantic memory caused by Alzheimer’s disease. Future work is needed for comparing MDS-based, feature-based, and hybrid models of similarity representation for the present types of everyday-object stimuli. Although our focus was on MDS in the present research project, and the MDS-based modeling yielded some useful results and insights, our collection of the similarity-judgment data provides an important and long-lasting contribution in and of itself. The results provide evidence that our proposed alternating-category approach to collecting similarity-judgment data from diverse everyday-object categories is a promising one. The similarity-judgment data should serve as a fertile database for researchers interested in testing alternative models of similarity and using the stimulus representations derived from such models as a source of input for computational models of cognitive processes.

## Data Availability

The datasets generated and analyzed during the current study are available at OSF website https://osf.io/aqb6w/. The experiments were not preregistered.

## Appendix

### Computing the AIC and BIC Statistics of MDS Model Fit

In general, the Bayesian Information Criterion (BIC; Schwarz, 1978) of model fit is given by

$$BIC=-2\ln L+P\cdot\ln(N),$$

whereas the Akaike Information Criterion (*AIC*; Akaike, 2003) is given by

$$AIC=-2\mathrm{lnL}+2\cdot P,$$

where ln*L* is the maximum ln-likelihood, *P* is the number of free parameters in the model, and *N* is the number of observations upon which the model fit is based. The latter terms in the BIC and AIC equations are penalty terms that penalize a model for its number of free parameters. The model that yields a smaller value of BIC or AIC is considered to provide a more parsimonious account of the data.

Following Lee (2001) and Tenenbaum (1996), minimizing the sum-of-squared deviations (SSD) between predicted and observed mean similarity judgments in a similarity matrix provides maximum-likelihood estimates of the values of the free parameters in the MDS model under the simplifying assumption that noise in each judgment cell is normally distributed with mean zero and constant variance. Following Lee (2001), the term -2ln*L* in the equations above is then given by

$$-2\ln L=\mathrm{SSD}/{\mathrm\sigma}_{\mathrm M^2},$$

where σ_M_^2^ is the pooled squared standard error of the mean across all cells of the similarity judgment matrices. Note that as the dimensionality of the MDS model increases, the SSD will decrease, but the penalty terms for BIC and AIC will increase because of the increasing number of free parameters (*P*) in the models.

In the present applications, because the similarity-judgment cell means are based on differing frequencies of observations (*f*), we use a frequency-weighted SSD measure and a frequency-weighted pooled squared standard error term. The weighted SSD is given by

$$\textrm{wSSD}=N\bullet \left[{\sum}_i\ \left({f}_i{\left({s}_i-{\hat{s}}_i\right)}^2\right)/{\sum}_i\ {f}_i\right]$$

where *f*_*i*_ is the frequency of observations in cell *i*; *s*_*i*_ is the observed mean similarity judgment in cell *i*; *ŝ*_*i*_ is the predicted mean-similarity judgment in cell *i*; and the sum is across all *N*=2,808 cells in the 36 similarity-judgment matrices. An analogous expression is used for computing the pooled frequency-weighted squared standard error term. The computed value was σ_M_^2^ = 0.0959.

In the case of a single *M*-dimensional Euclidean MDS solution for 13 items, there is a total of 13∙*M* coordinate parameters. However, the Euclidean space is translation-invariant, rotation-invariant, and reflection-invariant. As a consequence, *M*(*M*+1)/2 of these coordinate parameters can be set equal to zero without loss of generality—see Gronau and Lee (2020, p.326, Figure 3) for graphical illustrations of the consequences of these forms of spatial invariance on the parameter count. Thus, each individual-category MDS solution has 13∙*M* – *M*(*M*+1)/2 freely varying coordinate parameters. Finally, without loss of generality, the distance multiplier *v* in Equations 1 and 2 in the main text can be held fixed at 1 in the present modeling approach. Thus, in simultaneously predicting the data across all 36 categories, for an *M*-dimensional case the linear model uses a total of 36∙[13∙*M* – *M*(*M*+1)/2] + 1 free parameters and the exponential model uses a total of 36∙[13∙*M* – *M*(*M*+1)/2] + 2 free parameters.

The entries in Table 2 of the main text are based on the development provided above. We fitted the MDS models to the similarity judgment data by using the Hooke and Jeeves (1961) parameter-search algorithm. We used a variety of different starting configurations in the parameter searches, including starting configurations based on MATLAB’s MDSCALE, but the fits were essentially the same across the different starting configurations.

## Footnotes

1 Aggregated across all participants and trials, the proportion of responses at each similarity-judgment rating value were as follows: R1 = 0.073, R2 = 0.116, R3 = 0.156, R4 = 0.161, R5 = 0.122, R6 = 0.125, R7 = 0.120, R8 = 0.085, and R9 = 0.041. Hence, at the aggregate level, participants were somewhat less likely to respond with the extreme rating responses (1–2 and 8–9) than with the intermediate ones (3–7). This result seems intuitively sensible to us and suggests that in our experiment, participants tended to reserve extreme ratings for exceptional cases.

2 This function for relating the similarity ratings to distances in the space should be distinguished from Shepard’s (1987) classic proposal of a universal exponential law of generalization. Shepard’s proposal was that the probability that a learner would generalize a learned response from one stimulus to a novel one is exponentially related to the distance between the stimuli in psychological space. In the present case, the goal is to model similarity ratings, not probabilities of generalization. It is an open question what function is appropriate for capturing the relation between the ratings and derived psychological distances. If the to-be-predicted data were (normalized) generalization probabilities rather than ratings, then Shepard’s proposed law is a special case of Eq. 2 in which *b* = 0 and *u* = 1.

## Code availability

The computer code used for deriving the multidimensional-scaling solutions are available at OSF website https://osf.io/aqb6w/.
